# Schwannoma of the radial nerve: a case report

**DOI:** 10.11604/pamj.2022.43.139.37453

**Published:** 2022-11-14

**Authors:** Jihane Hamdaoui, Hind Elkamch, Noureddine Gharib, Samir El Mazouz, Abdellah Abbassi, Jawad Hafidi

**Affiliations:** 1 Plastic Surgery Department, Ibn Sina University Hospital of Rabat, Rabat, Morocco

**Keywords:** Schwannoma, radial nerve, delayed diagnosis, enucleation, case report

## Abstract

Schwannomas are the most common benign tumors of the peripheral nerves, but represents only 5%-8% of all soft tissue tumors. Their diagnosis is usually delayed due to their slow growth and the nervous adaptation to their increased volume. Ultrasound sonography and magnetic resonance imaging (MRI) images usually ease the diagnosis. Correct enucleation offers very good postoperative outcomes and avoids recurrences. We report an unusual case of schwannoma of the radial nerve (RN) that remained asymptomatic for one year and a half, and treated well, had good outcomes.

## Introduction

Schwannomas are the most common benign tumors of the peripheral nerves, composed exclusively of Schwann cells derived from the neural crest. They are solitary in 90% of the cases, occasionally there can be multiple lesions or a lesion associated with neurofibromatosis. The diagnosis is usually delayed due to their slow growth and the nervous adaptation to their increased volume [1]. We report an unusual case of schwannoma of the radial nerve located on the external face of the lower third of the left arm that remained asymptomatic for one year and a half.

## Patient and observation

**Patient information:** a 20-year-old male patient, non-smoker, non-alcoholic with no particular medical or family history.

**Clinical findings:** the clinical examination found an ovoid tumefaction of the external face of the lower third of the left arm, measuring 5 cm in length, solid, freely mobile. Percussion over the mass produced Tinel's-like paresthesia in the radial nerve territory. The neurological examination showed preserved motor and sensory functions. The rest of the exam was normal.

**Timeline of current episode:** the beginning of his disease goes back to a year and a half before his admission by the occurrence of a swelling of the external face of the lower third of the left arm gradually increasing in volume and causing dysesthesia on contact (electric shocks) with an evolution marked by motor discomfort after repeated movements of the limb.

**Figure 1:**
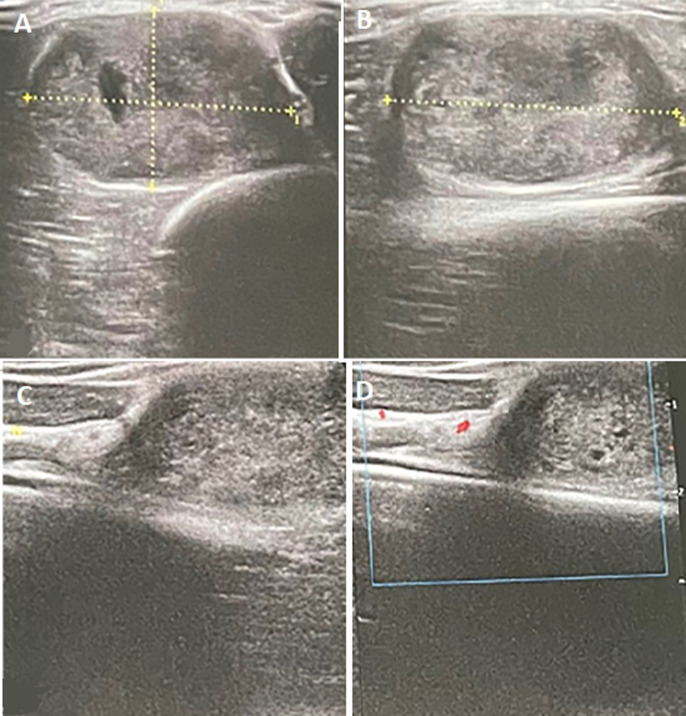
ultrasound aspects; A,B) measures of the mass 27mm/30mm/19mm; (C,D) ultrasound showing a fusiform, homogeneous and well-defined mass in relation to the course of the radial nerve

**Figure 2:**
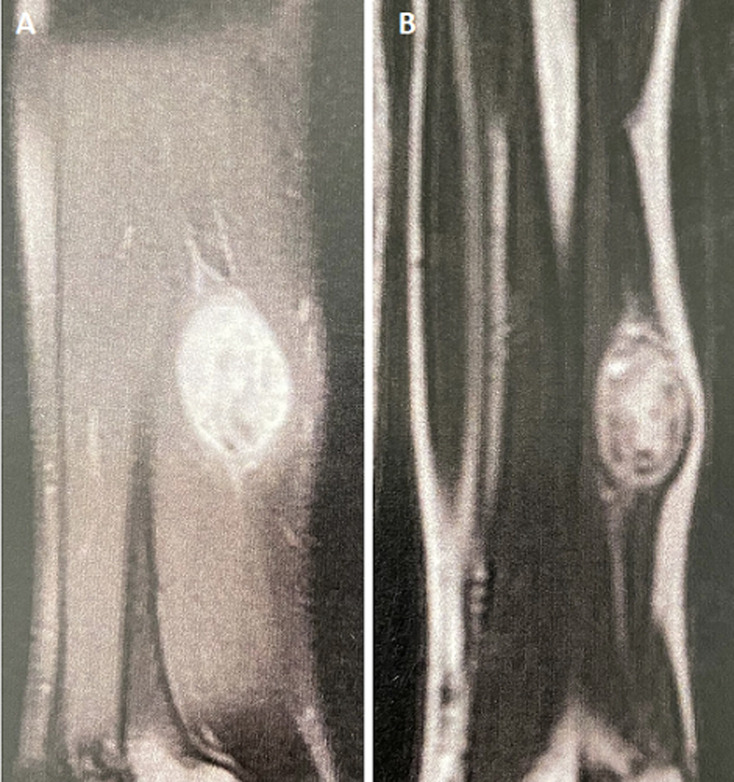
A,B) magnetic resonance imaging (MRI) aspect: soft tissue process well delimited and pseudo-encapsulated in relation to the course of the radial nerve compatible with a schwannoma

**Diagnostic assessment:** soft tissue ultrasound revealed a fusiform mass, homogeneous, well defined and hypoechoic resting on the biceps muscle with no sign of invasion or rupture of its fibers, measuring 27mm/30mm/19mm (Figure 1). The MRI objectified a tissue process of 35/25/43 mm, which had hyposignal on T1, hypersignal on T2, well delimited and pseudo-encapsulated, eccentric in relation to the course of the radial nerve (Figure 2).

**Diagnosis:** the results were consistent with schwannoma of the radial nerve.

**Figure 3:**
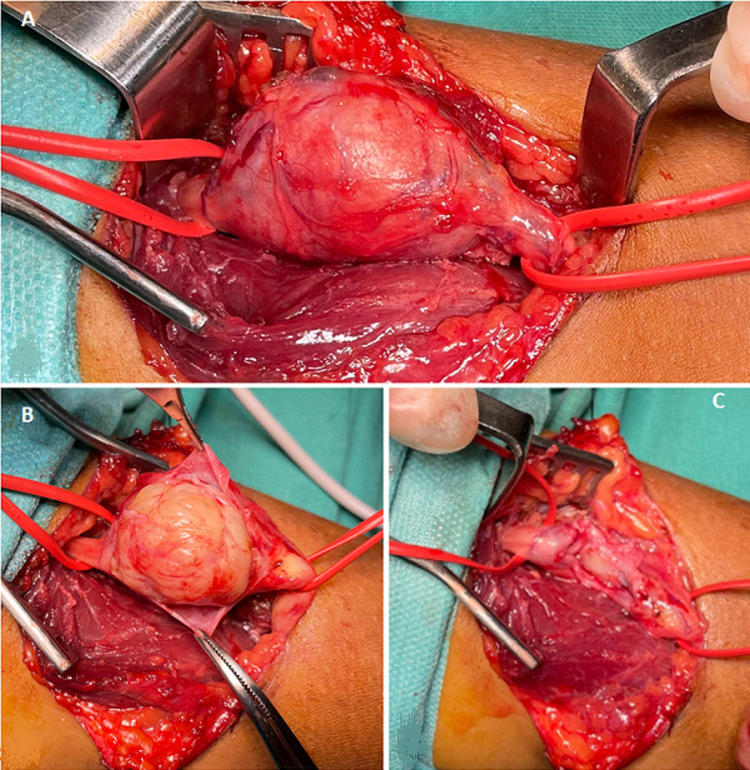
surgical exploration: A) encapsulated tumor sitting between the fibers of the biceps muscle on the path of the radial nerve; B) enucleation without fascicular lesion; C) aspect of the radial nerve after the enucleation

**Therapeutic interventions:** the treatment was surgical: under axillary brachial plexus block, A Z incision centered on the tumor was performed, exploration revealed an encapfiguresulated tumor sitting between the fibers of the biceps muscle on the path of the radial nerve, an enucleation was performed without fascicular lesion (Figure 3). Histological exam of the tumor showed non nucleated fibrillar areas lined by a palisade of Schwann cell nuclei characteristic of a benign schwannoma.

**Follow-up and outcome of interventions:** postoperatively, the patient was seen after 2 days, the preoperative paresthesia had completely disappeared, a cramp-type motor disorder of the last two fingers was noted with disappearance after one week. At a 12 months follow-up, there has been no recurrence and the patient was very satisfied.

**Patient perspective:** “it took me so much time to figure out that I have a mass, I can´t believe the pain is gone, now I can live normally again thanks to you”.

**Informed consent:** written consent for publication was obtained from the patient.

## Discussion

Schwannomas (also known as neuromas, neurinomas “of Verocay” and neurilemmomas) are benign, well-encapsulated, slow-growing nerve sheath tumors composed exclusively of Schwann cells derived from the neural crest [2,3]. They are the most common benign tumors of the peripheral nerves but represents only 5%-8% of all soft tissue tumors [1]. The tumor can originate from any myelinated central or peripheral nerve with Schwann cells. The World Health Organization classifies schwannoma as a grade I benign tumor. Schwannomas are solitary in 90% of the cases. Multiple tumors in the same patient should bring attention to syndromic associations (neurofibromatosis type 2, schwannomatosis, and Carney complex) [4-7]. They most commonly occur in adults between 20 and 50 years of age, without distinction of gender, with an approximate one sex ratio [8]. Schwannomas can be asymptomatic or can produce pain, a positive Tinel´s sign or a Tinel´s-like sensation, and sensory alterations. The slow growth pattern of benign nerve tumors allows for adaptation of the nerve function to the pressure effects [9].

The ultrasound sonography and MRI tests play an important role in guiding preoperative diagnoses, since they do not have 100% accuracy. On ultrasound, schwannomas are usually seen as homogeneous, well-defined hypoechoic masses, often ovoid, and can show the origin and relationship of the tumor with the affected nerve. MRI can provide useful information about morphological data on the tumor; however, it cannot provide dynamic information [10]. Although low-intense signals on TI-weighted images and hyperintense signals on T2-weighted images are common findings of schwannomas [8], MRI also give useful information regarding tumor extent, anatomical location, tumor size, and relationship of peripheral nerve, and for appropriate planning of surgical therapy and preoperative diagnosis [11].

Surgical excision is the treatment of choice. Schwannomas are theoretically removable because of their eccentric, noninfiltrating growth, thus allowing their enucleation without or with only slight damage to fascicular structure [12,13]. Some authors showed that the size of the tumor, longer history, or presence of preoperative neurological symptom correlated with the incidence of neurological deficit; hence, is recommended to do early excision to have better clinical outcome and to avoid postoperative neurological deficits [14].

In our case, it's a schwannoma located on the radial nerve occurring in a 20-year-old male patient with a delayed diagnosis to one year and a half, causing swelling and a Tinel's-like paresthesia, the diagnosis was made on ultrasound and MRI, as the diagnosis of these tumors is often delayed, and sometimes done too late when the neurological deficit is irreversible. Hence, the early and the correct enucleation without injuring nerve's fascicules has better clinical outcome and avoids postoperative sequels.

## Conclusion

Schwannomas are benign tumors, that have low incidence, slow growth and atypical symptoms often leading to misdiagnosis or delayed diagnosis, as in our reported case. Ultrasound sonography and MRI images usually ease the diagnosis. Correct enucleation offers very good postoperative outcomes and avoids recurrences. The patient in this case report had an excellent clinical outcome and no recurrence at 12 months follow-up.
